# Mate-pair sequencing assisted prenatal counseling for a rare complex chromosomal rearrangement carrier

**DOI:** 10.1093/hmg/ddaf012

**Published:** 2025-03-05

**Authors:** Lu Wan, Zeng Baitao, Tan Yuxin, Chen Zhongfa, Zhou Jihui, Huang Ning, Yang Bicheng, Huang Shuhui, Liu Yanqiu, Yuan Huizhen

**Affiliations:** Medical Genetic Center, Jiangxi Provincial Key Laboratory of Birth Defect for Prevention and Control, Jiangxi Maternal and Child Health Hospital, #508 Xizhan Street, Honggutan District, Nanchang, Jiangxi 330006, China; Medical Genetic Center, Jiangxi Provincial Key Laboratory of Birth Defect for Prevention and Control, Jiangxi Maternal and Child Health Hospital, #508 Xizhan Street, Honggutan District, Nanchang, Jiangxi 330006, China; Maternal and Child Health Affiliated Hospital of Nanchang University, #461 Bayi Road, Donghu District, Nanchang, Jiangxi 330006, China; Medical Genetic Center, Jiangxi Provincial Key Laboratory of Birth Defect for Prevention and Control, Jiangxi Maternal and Child Health Hospital, #508 Xizhan Street, Honggutan District, Nanchang, Jiangxi 330006, China; Medical Genetic Center, Jiangxi Provincial Key Laboratory of Birth Defect for Prevention and Control, Jiangxi Maternal and Child Health Hospital, #508 Xizhan Street, Honggutan District, Nanchang, Jiangxi 330006, China; Medical Genetic Center, Jiangxi Provincial Key Laboratory of Birth Defect for Prevention and Control, Jiangxi Maternal and Child Health Hospital, #508 Xizhan Street, Honggutan District, Nanchang, Jiangxi 330006, China; Medical Genetic Center, Jiangxi Provincial Key Laboratory of Birth Defect for Prevention and Control, Jiangxi Maternal and Child Health Hospital, #508 Xizhan Street, Honggutan District, Nanchang, Jiangxi 330006, China; Medical Genetic Center, Jiangxi Provincial Key Laboratory of Birth Defect for Prevention and Control, Jiangxi Maternal and Child Health Hospital, #508 Xizhan Street, Honggutan District, Nanchang, Jiangxi 330006, China; Medical Genetic Center, Jiangxi Provincial Key Laboratory of Birth Defect for Prevention and Control, Jiangxi Maternal and Child Health Hospital, #508 Xizhan Street, Honggutan District, Nanchang, Jiangxi 330006, China; Medical Genetic Center, Jiangxi Provincial Key Laboratory of Birth Defect for Prevention and Control, Jiangxi Maternal and Child Health Hospital, #508 Xizhan Street, Honggutan District, Nanchang, Jiangxi 330006, China

**Keywords:** complex chromosomal rearrangement, mate-pair sequencing, prenatal diagnosis, genetic consulting

## Abstract

**Objective:**

This study was aimed to identify a rare complex rearrangement and assist prenatal counseling.

**Method:**

Mate-pair sequencing (MPseq) combined with karyotypes, copy number variants sequencing and whole exome sequencing was used to provide accurate chromosome breakpoints and assist prenatal diagnosis for a mentally retarded pregnant woman.

**Result:**

MPseq indicated a complex rearrangement involved 25 breakpoints and fusions, disrupting 6 genes. Among which, *ZMIZ1* was associated with neurodevelopmental disorders with dysmorphic facies and distal skeletal abnormalities, which was consistent with the phenotype of pregnant women.

**Conclusion:**

MPseq was a cost-effective and accurate method that could be used as a complementary tool for human genetic diagnosis and prenatal counseling.

## Graphical Abstract

**Graphical Abstract:**
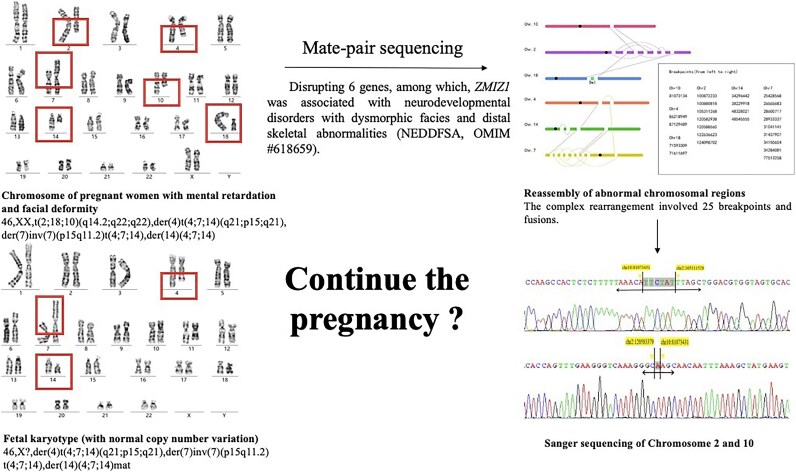

## Introduction

Complex chromosomal rearrangements (CCRs) are described as structural rearrangements involving at least three cytogenetic breakpoints on more than two chromosomes [1]. Balanced CCRs usually have no loss of genetic information and normal phenotype, but are closely associated with adverse pregnancy outcomes such as recurrent miscarriage, stillbirth, mental retardation and other congenital malformations [2, 3]. Male carriers of CCR may have decreased fertility and usually present with azoospermia or oligospermia [4, 5]. For female carriers, complex rearrangements involving certain autosomal structural abnormalities may lead to ovarian dysfunction or early-onset ovarian insufficiency [6]. CCR carriers have lower probability of forming normal gametes and higher probability of adverse pregnancy outcomes than those with reciprocal translocations. The more complex, the higher risk of gametic imbalance, hence the higher risk of producing affected offspring [7]. Thus, precise diagnosis and characterization of CCRs are very important.

G-banding karyotyping is the most regular test used to detect apparent chromosomal rearrangements, molecular cytogenetic techniques such as fluorescence *in situ* hybridization (FISH), chromosomal microarray analysis (CMA) could also be used, but they all have certain limitations [8–10]. Mate-pair sequencing (MPseq) takes advantage of a unique library preparation chemistry including the cyclization of long DNA fragments, allowing unique paired end sequencing applications. This method improves the accuracy of structural variation and copy number detection including some recessive and complex rearrangements that cannot be detected by conventional cytogenetic methods [11, 12]. In this study, MPseq was applied to identify a rare complex rearrangement and provide accurate breakpoints, thus assisted prenatal counseling.

## Results

### Chromosome karyotype analysis

**Figure 1:**
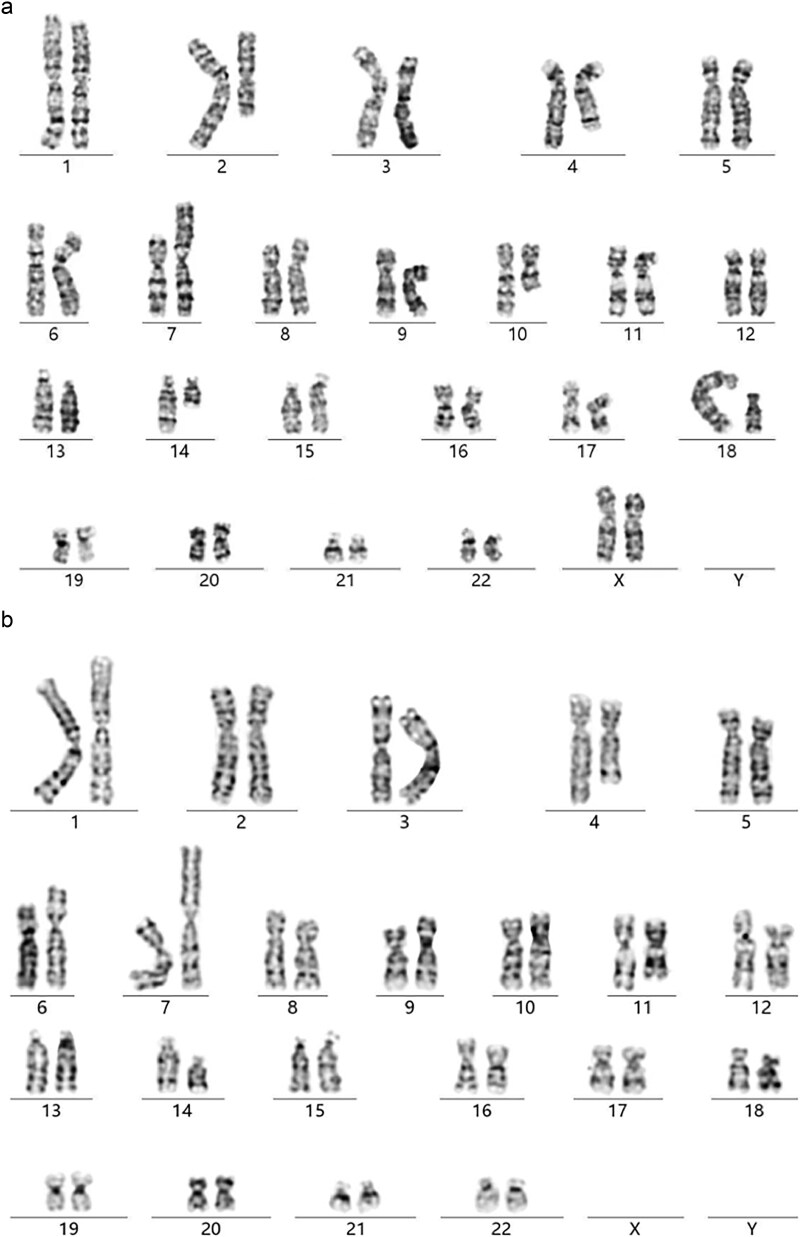
Maternal and fetal amniotic karyotype. (a) The maternal karyotype (b) Fetal karyotype.

The karyotype of the pregnant woman involving six chromosomes was described as 46,XX,t(2;18;10)(q14.2;q22;q22),der(4)t(4;7;14)(q21;p15;q21),der(7)inv(7)(p15q11.2)t(4;7;14),der(14)(4;7;14). While the fetal karyotype was 46,X?,der(4)t(4;7;14)(q21;p15;q21),der(7)inv(7)(p15q11.2)t(4;7;14),der(14)(4;7;14)mat. According to relative laws and regulations, the fetal sex chromosome was hidden (Fig. 1).

### Mate-pair sequencing analysis

**Figure 2:**
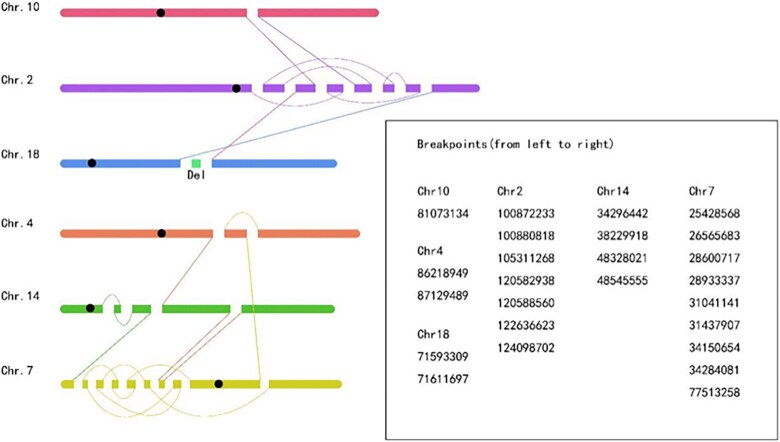
Reassembly of abnormal chromosomal regions.

MPseq indicated a far more complicated rearrangement. A microdeletion on chromosome 18 (Chr18:71593309-71611697) and a complex chromosomal rearrangement were found involving chromosome 2, 4, 7, 10, 14, and 18. Chromosomes 2, 7 and 14 were not only involved in translocations, but also had inversions, especially there were many intricate position exchanges between chr2 and chr7. The complex rearrangement involved 25 breakpoints and fusions. Variations and breakpoints were showed in Fig. 2.

### Sanger sequencing of chromosome 2 and 10

**Figure 3:**
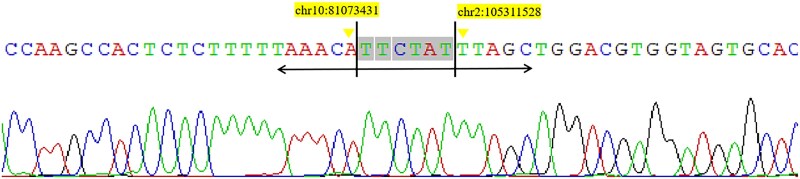
Link1(grch37:chr10:81073431-TTCTAT-grch37:chr2:105311528).

**Figure 4:**
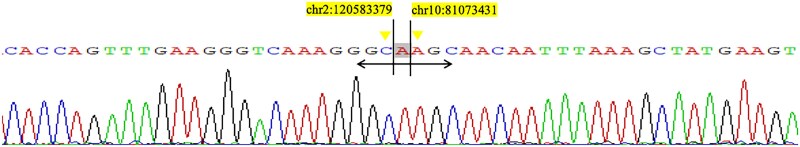
Link2(grch37:chr2:120583379-A-grch37:chr10:81073431).

The Sanger sequence chromatogram showed that the nucleotide 81 073 431 of chromosome 10 was connected to the nucleotide 105 311 528 of Chromosome 2 by TTCTAT bases (Fig. 3). The Sanger sequence chromatogram of the other side showed that the nucleotide 81 073 431 of chromosome 10 was connected to the nucleotide 120 583 379 of Chromosome 2 by A bases (Fig. 4). The connection area of the chromosome 2 and 10 was found by Sanger sequencing.

## Discussion

In this study, we presented a rare prenatal consultation of a mentally retarded woman with complex structural chromosomal abnormalities. In order to determine whether the pregnant women have copy number variants (CNVs) and gene mutations associated with intelligence, whole exome sequencing (WES) and CNV-seq were performed. Analysis of pathogenic genes identified in OMIM database showed that no pathogenic gene variation was found, and CNV was normal.

Since the pregnant women showed intellectual retardation, it was reasonable to speculate that may be related to the genetic variation of non-coding regions caused by complex balanced translocations. MPseq can identify almost all hidden recessive chromosomal abnormalities or complex rearrangements of the genome without obtaining the cytogenetic information of the patient and can characterize translocation breakpoints at the nucleotide level, providing accurate breakpoint sequences for subsequent studies, which is of great value in guiding eugenics and fertility [13, 14]. It was found that pregnant women had complex balanced chromosomal translocations, involving 25 chromosome breaks and fusions, disrupting 6 genes (*ZMIZ1*, *PTPN4*, *MAPK10*, *CREB5*, *BMPER*, *PHTF2*) break rearrangement. Among which, *ZMIZ1* was associated with neurodevelopmental disorders with dysmorphic facies and distal skeletal abnormalities (NEDDFSA, OMIM #618659).

NEDDFSA is a rare syndromic disorder characterized by global neurodevelopmental delay, hypotonia, poor overall growth, poor speech/language ability [15]. There is evidence that heterozygous mutations in the *ZMIZ1* gene on chromosome 10q22.3 can lead to NEDDFSA [16]. The mental development of NEDDFSA patients varies from severe inability to speak to mild ability to attend special schools. In 2015, a girl with intellectual disability and neuropsychiatric symptoms was reported with a *de novo* balanced translocation, t(10;19)(q22.3;q13.33), that resulted in gene fusion between *ZMIZ1* (chr10) and *PRR12* (chr19), thereby disrupting the zinc-finger motif of *ZMIZ1* [17]*.* In 2019, 19 subjects with intellectual disability and developmental delay were reported carrying variants in *ZMIZ1*, 2 subjects had a balanced translocation disrupting *ZMIZ1* or involving a regulatory region of *ZMIZ1* [15]. Lately, a *de novo* missense variant (c.2330G > A, p.Gly777Glu, G777E) was identified in the exon 20 of *ZMIZ1*, which was first discovered in a Chinese female with NEDDFSA [18]. Considering that carriers of complex balanced translocations had low probability of producing normal gametes, the fetus was not involved in chromosome 10 abnormalities and had a lower risk of developing related diseases. After adequate genetic counseling, the pregnant woman decided to continue the pregnancy. Fortunately, with follow-up, no skeletal dysplasia and developmental delay were found in the baby.

Most apparently CCRs could be detected by karyotyping, but its resolution was limited to 5~10 Mb and could not provide accurate breakpoints [19]. Accurate breakpoints mapping was the key to providing reproductive risk prediction, genetic counseling, and fertility guidance for couples with CCRs [20]. MPseq approach could assist reproduction for carriers with recurrent miscarriage due to chromosomal abnormalities. In 2020, Jian Ou successfully combined MPseq and preimplantation genetic testing to help a couple [karyotyped as 46, XX, der(1)t(1;4)(p22;q31.1),der(4)ins(5;4)(q22;q25q28)t(1;4),der(5)ins(5;4)] have a healthy child [21]. A research data showed that, compared with conventional karyotype analysis, MPseq significantly improved the detection rate of chromosomal abnormalities (11.7%) [22].

With the development of genomics, optical genome mapping (OGM) as a new method that could accurately detect structural variations with a high resolution and provide the breakpoint regions at molecular level [23]. Rao *et al* successfully detected additional CCRs and balanced translocations through OGM, further clarifying the underlying genetic causes of recurrent spontaneous abortions [24]. Yang presented a rare familial CCR involving three chromosomes and four breakpoints, and provided precise and detailed information for the subsequent reproductive decision-making and genetic counseling of the patient by OGM [25]. However, the cost of OGM was higher than MPseq, leading MPseq to be reported as a highly accurate, cost-effective approach. Besides the application of detecting chromosome variations, MPseq was also being used to uncover novel pathogenic gene fusions in leukemia [26, 27]. However, it still has limitations, in areas near the centromere or telomere or large fragments of many similar repeats, which are generally difficult for NGS to detect [28]. In addition, MPseq cannot reliably detect structural rearrangements of less than 10% [29, 30]. Making it could be a complementary tool for human genetic diagnosis. For couples with chromosomal abnormalities who need prenatal diagnosis, the detection of fetal karyotype, CNV and MPSeq at the same time can speed up the diagnosis and provide timely, scientific and reasonable fertility guidance. And for patients with chromosomal abnormalities with a clinical phenotype, it can be recommended to perform MPseq to detect whether the genes of the related diseases are interrupted.

## Conclusions

MPseq is capable of identifying and characterizing chromosomal structural variations, providing valuable insights for disease diagnosis. In this paper, we demonstrated the feasibility of mate-pair sequencing analysis to improve the detection of chromosomal structural variants and prediction of genotypic and phenotypic outcomes, which is a cost-effective and accurate method. It can play a vital role in assisting a rare complex balanced translocation carrier to give birth to a healthy baby.

## Methods

### Case presentation

**Figure 5:**
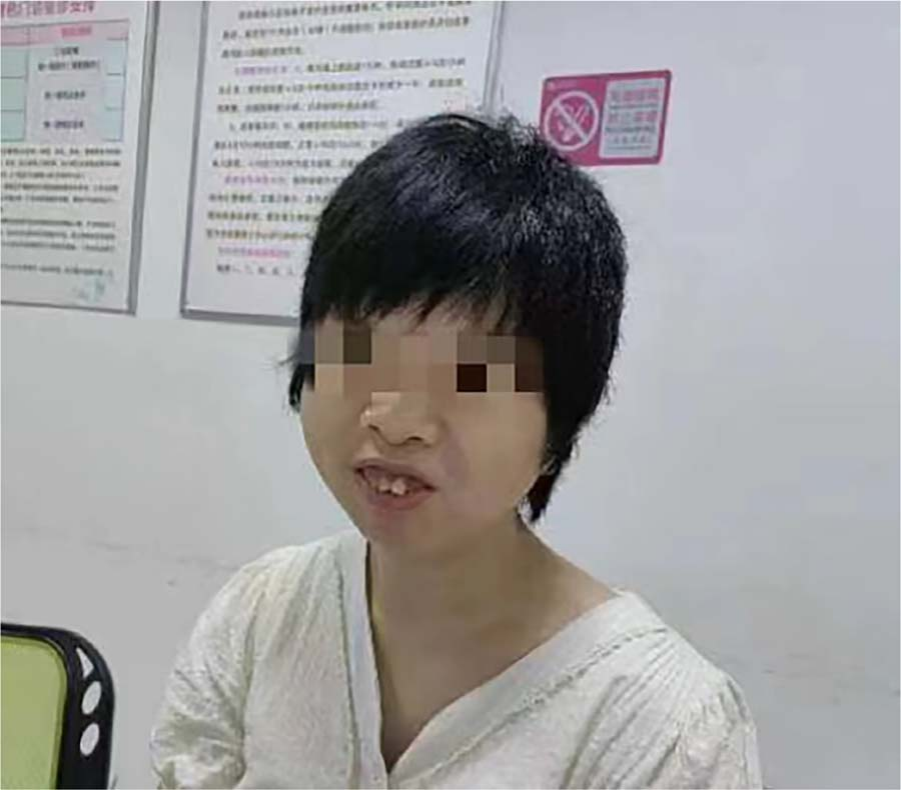
The appearance of the pregnant woman, with protruding upper jaw and disordered upper teeth.

A pregnant woman with mental retardation experienced two early miscarriages. She had a protruding upper jaw, disordered upper teeth, low nose bridge, slightly wider eyes, low back hairline, and with no family history of genetic disease (Fig. 5). The family came to our hospital to inquire if the fetus would inherit mental retardation. Karyotyping was performed using G-banding at the 400–550 level on cultured lymphocyte metaphases. Her husband had normal karyotype (46,XY), while she was found to carry complex translocation and inversion, with the karyotype 46,XX,t(2;18;10)(q14.2;q22;q22),der(4)t(4;7;14)(q21;p15;q21),der(7)inv(7)(p15q11.2)t(4;7;14),der(14)(4;7;14). To determine whether the fetus will inherit this complex structural variations, and assess the risk of future growth and related diseases, amniocentesis was performed at 20 weeks’ gestation. Informed consent was signed for all tests.

### Karyotype

2 ml of peripheral blood was taken with heparin anticoagulation. Amniotic fluid samples were collected by ultrasound-guided transabdominal amniocentesis. Cells were cultured and prepared for G-banding karyotyping using standard protocols [31].

### Low-coverage whole genome sequencing

To further verify the results of karyotype, we adopted mate-pair sequencing method [32]. The genomic DNA(1 μg) was extracted and the concentration was detected by Qubit. The qualified genome DNA were used to construct a non-size selected mate-pair library by MP Library Prep Kit (GeneTech Co., Ltd, Shanghai, China) and then subjected to 100-bp-end sequencing by DNBSEQ-T7RS platform (MGI Technology Co., Ltd, Shenzhen, China) and a target mean coverage of > 5-folds. We can use uniquely paired reads to find all chromosome CNVs and structural variants (SV), as well as corresponding breakpoints across the genome, and the accuracy of the breakpoints could be accurate to a small region of ±500 bases. Finally, the Sanger sequence is verified precisely for the breakpoint.

### Verification by sanger sequencing

To make sure the exact location of the breakpoint, two primers were designed using Primer 5 and synthesized to amplify the connection area of the chromosome 2 and 10. The primers were as follows:

Link1F,5′-GCAGCCCTCTCAGAACAGAG-3′;

Link1R,5′-AAGGTCAGCCAGGTCAGTTG-3′;

Link2F,5′-TGGGTCCCAACACAGACCTA-3′;

Link2R, 5′-GGAAGTCTTGGAGAGGTGGC-3′.

The PCR amplified procedure was performed as described by Zeng [33]. The PCR products were sequenced by a sequencing provider. Mapping and aligning the sequencing reads to reference genomes and was completed by SeqMan Pro.
